# Mindful With Your Baby/Toddler: A Single Case Design (SCD) Study

**DOI:** 10.1177/01632787241297966

**Published:** 2024-11-11

**Authors:** Mirla A. Schaeffer, Eva S. Potharst

**Affiliations:** 1 UvA Minds, Academic Outpatient (Child and Adolescent) Treatment Center of the University of Amsterdam, The Netherlands; 2 Amsterdam Law and Behaviour Institute (A-LAB), 1190 Vrije Universiteit Amsterdam, The Netherlands; 3 4494 Netherlands Institute for Crime and Law Enforcement (NSCR), The Netherlands; 4 Research Institute of Child Development and Education, University of Amsterdam, The Netherlands

**Keywords:** babies, toddlers, mindfulness, mindful parenting, parental stress, parental self-efficacy, maternal internalizing problems, case-based design

## Abstract

Transitioning to motherhood comes with new and intensive tasks that may cause parental stress, low parental self-efficacy, and internalizing problems. This can in turn negatively affect the mother-child relationship. Mindful with your Baby/Toddler (MwyB/T) is a mindfulness-based intervention for parents of young children experiencing parental stress and internalizing problems. Previous evaluative studies showed promising results, but methodology of these studies was limited. The current study used a single case design, including a baseline, intervention, posttest, and follow-up phase, to evaluate the effectiveness of MwyB/T. Ten participants were included and completed daily administered personalized items and validated questionnaires measuring mindfulness, mindful parenting, parental self-efficacy, internalizing problems, and parental stress, for 10 participants. Personalized items were first coded into themes and then assessed using visual analysis and descriptive effect size measures. Reliable change indices were computed for the questionnaires. All mothers improved on personalized items, with most improving on most (or all) of their items. On the questionnaires the majority of mothers improved. Results indicate that MwyB/T could benefit mothers with various intervention goals. More research is needed on the role of personalized items, both as a research measure and an as a possible additional element of interventions.

The postpartum period, defined as the first year after birth, is often considered a period filled with happiness (Eckstein et al., 2019). However, the transition to motherhood also comes with new and intensive tasks on a physical, emotional, and social level (Dol et al., 2019). Examples are learning to adapt tobodily changes and changes in the partner relationship, forming a mother-identity, and learning to mother (Drozdowskyj et al., 2020; Nelson, 2003; Nonnenmacher et al., 2016). These tasks and changes can be challenging to new mothers, cause parental stress, and increase their vulnerability to internalizing problems (Fernandes et al., 2020), such as depressive symptoms and anxiety. Although this is true for both mothers and fathers, mothers do appear to be at greater risk for postpartum parental stress and internalizing problems (Epifanio et al., 2015; Vismara et al., 2016). Prevalence rates of depression in the first year after birth (postpartum depression; Shorey et al., 2018) worldwide range from 6% to 47% (Wang et al., 2021). Prevalence of postpartum depression in the Netherlands (where the current study was conducted) is about 11% (Wang et al., 2021). Furthermore, prevalence rates for maternal postpartum anxiety range from 4% to 39% (Goodman et al., 2016).

Successful transitions to motherhood have positive, long-term effects on maternal warmth and children’s emotion regulation (Richter et al., 2021). Parental stress and internalizing problems are negatively associated with mothers’ parental self-efficacy (Goodman et al., 2016; Leahy-Warren & McCarthy, 2011), which is defined as “expectations parents hold about their ability to parent successfully” (Jones & Prinz, 2005, p. 342). In turn, parental self-efficacy is an important predictor of parental competence and child functioning (Jones & Prinz, 2005). Also, maternal postpartum internalizing problems are associated with an impaired mother-infant bonding (Dubber et al., 2015; Goodman et al., 2016; Moehler et al., 2006). Further, parental stress and postpartum internalizing problems are related to child social-emotional problems (Frederiksen et al., 2019; Goodman et al., 2016; Stein et al., 2014). Finally, untreated postpartum internalizing problems can even lead to infanticide and suicide (Kendig et al., 2017).

Moving a step forward on the path of motherhood, toddlerhood too is a time associated with distinctive demands, tasks, and challenges placed on mothers (Kwon et al., 2013). These are related to the toddler’s high need for autonomy and independence combined with a high need for support in behavior and emotion regulation (Bechtel-Keune et al., 2016). Challenges toddlers’ mothers encounter are associated with parenting stress (Kwon et al., 2013). Maternal depression prevalence remains high during toddlerhood. Approximately 15% of mothers score above the clinical cut-off on the Edinburgh Postnatal Depressive Scale, both two and a half and four years postpartum (Johansson et al., 2020; Woolhouse et al., 2016). Moreover, both symptoms of depression and anxiety in early parenthood predict parental stress in parents with toddlers (Saisto et al., 2008). Parental stress in its turn is an important predictor of both child emotional and behavioral problems, poorer mother-infant dyadic interactions, and maternal depression during this period (Crnic et al., 2005; Johansson et al., 2020; Woolhouse et al., 2016). Given the negative consequences parental stress, low parental self-efficacy, and maternal internalizing problems can have during the postpartum period and toddlerhood, it is important to intervene in an effective way. The current study will investigate the effectiveness of Mindful with your Baby/Toddler (MwyB/T; Potharst et al., 2017; Potharst, Zeegers, & Bögels, 2021), a parenting intervention based on the principles of mindfulness and mindful parenting.

Mindfulness can be defined as “paying attention in a particular way: on purpose, in the present moment, and non-judgmentally” (Kabat-Zinn, 1994, p. 4). Mindfulness interventions are effective in improving stress regulation by decreasing emotional reactivity and increasing cognitive reappraisal (Khoury et al., 2015). Mindful parenting interventions are used to improve co-regulation in parent-child dyads (Duncan et al., 2009). Mindful parenting has been described as “a present-moment awareness to […] parenting that includes listening with full attention, bringing emotional awareness and non-judgmental acceptance to their parenting interactions, and practicing self-regulation and compassion in their parenting relationship” (Duncan et al., 2009, p. 266). Mindfulness during pregnancy may be a protective factor for parental stress three years later (Boekhorst et al., 2020). Practicing mindful parenting allows parents to be aware in the present-moment, while viewing their current experiences with their child in the context of the long-term relationship with them (Duncan et al., 2009). By increasing their self-regulation parents experience less over-reactivity or stress (Kil & Antonacci, 2020), and are able to respond consciously to their child’s needs. Mindful parenting has been found to be related to observed parenting and parent-child interaction (Potharst, Zeegers, & Bögels, 2021) and mindful parenting-based interventions have been found to decrease parental stress, increase parental self-efficacy (depending on improvements in mindful parenting), and reduce maternal internalizing problems (depending on improvements in mindfulness; Bögels et al., 2013; Corthorn, 2018; Potharst, Kuijl, et al., 2022; Perez-Blasco et al., 2013).

MwyB/T is a group training for parents of young children experiencing mental health problems and/or parenting related stress or insecurity. During the training mothers learn to be aware of their inner experiences in the present moment. Because the children are present during a large part of the training, the mothers can practice mindfulness in relation to their child. They learn to be attentive to their child and the signals the child shows, and to apply mindfulness in stressful situations. Previous studies have shown promising results on a wide variety of both mother and child outcomes. Studies investigating the effects of MwyB found a significant improvement in mindfulness and mindful parenting (medium to large effects), in parental stress (small to medium effects), parental self-efficacy (small to medium effects) and in maternal internalizing problems (medium effects) (Potharst et al., 2017, Potharst, Kuijl, et al., 2022). In a non-clinical setting, improvement was found on mindfulness (large effects), parental stress (small to medium effects), and maternal internalizing problems (medium effects; Potharst, Veringa-Skiba, et al., 2022). In a non-randomized study with waitlist control, investigating the effects of MwyT, significant improvement was found in mindfulness, mindful parenting, parental stress, parental self-efficacy, and in maternal internalizing problems (all moderate to large effects; Potharst, Zeegers, & Bögels, 2021).

However, the level of evidence that these studies provided is modest, due to limitations in the methodology that was used. One study was non-experimental (using a longitudinal design with a pretest, posttest, en two follow-up measurements; Potharst et al., 2017). The other studies were quasi-experimental, using a waitlist assessment to control for the effects of time and assessment (Potharst, Kuijl, et al., 2022, Potharst, Veringa-Skiba, et al., 2022). For future research, performing a randomized controlled trial (RCT) has been advised to decrease the risk of bias (Potharst, Veringa-Skiba, et al., 2022). However, whilst RCTs are seen as the gold standard in effectiveness research, the design also has some disadvantages (Sanson-Fischer et al., 2007). An important practical restriction of RCTs is the requirement of large participant numbers. This makes them require high monetary, time, and personnel resources that are often not available to researchers, especially in clinical settings (Sanson-Fischer et al., 2007). Further, the use of specific control conditions is not always possible due to a large heterogeneity in participants’ problems: MwyB/T can be indicated both for varying parental mental health problems, for problems in the interaction between parent and child and for child regulation problems, while most interventions that are available for this age group have a more narrow indication area. Comparing MwyB/T with treatment as usual such as offered in specialized mental health care may be an ethical option, but treatment as usual could differ between participants. Another possibility would be to use a waitlist condition as a control condition in an RCT. However, assigning the target audience of MwyB/T to a waitlist control group would mean withholding care, which could lead to a deterioration of problems in a critical period. Furthermore, at a statistical level, in RCTs results are generally depicted in terms of group means and standard deviations. This entails that as long as the intervention group as a whole appears to benefit compared to the control group the intervention is regarded generally effective, even if in reality only a part of the intervention group responded to the treatment (Lefkowitz & Jefferson, 2014). For example, RCTs most often do not reveal whether ethnic minorities or individuals of lower socio-economic status also benefit from a mindfulness-based intervention (Waldron et al., 2018).

A recent meta-analysis investigating the effectiveness of psychotherapy in decreasing depressive symptoms in adults (Cuijpers et al., 2021) demonstrates that even when the group mean score may improve, individuals may not always show improvement. In this meta-analysis, a response was defined as a 50% reduction in depressive symptomology. Only 41% of the participants, compared to 16% for those in waitlist conditions, responded to that extent (Cuijpers et al., 2021). Further, only approximately one third of participants remitted after psychotherapy (Cuijpers et al., 2021). This means that more than half of participants do not respond to psychotherapy and two-thirds are still suffering from (mild) depression after treatment. Similarly, a randomized controlled trial investigating the effectiveness of a brief online parenting intervention (Baker et al., 2017) found clinically improved child behavior subscale scores for 44%–55% of their participants and clinically improved parenting subscale scores for 52%–70% of the participants. Looking at reliable change they found improvement in child behavior for 38%–41% of the participants dependent on the subscale, and for only 14%–28% of the participants they found subscale improvements in parenting. Finally, a meta-analysis on the effectiveness of another parenting intervention (Leijten et al., 2018) found that 40.1% of the children scoring above the 90^th^ percentile on parent-reported conduct problems at baseline, remained at this level at posttest. Further, 56% of parents scoring above the cut-off for depression at baseline, continued to score above cut-off at posttest. Again, this means that while these (parenting) interventions may be effective for part of the population, for a substantial other part they are not.

Single-case design (SCD) is a method to analyze change in a variable for individual participants over time and between study phases. A SCD is performed by collecting datapoints at consistent intervals during all phases and statistically compare phases. SCDs can be a viable, scientifically rigorous alternative to RCTs, not only to assess whether, but also for whom psychological and behavioral interventions work (Borkardt et al., 2008; Borkardt & Nash, 2014; Lobo et al., 2017; Smith, 2012). First of all, SCDs require smaller samples and fewer resources (Kratochwill et al., 2010), making them more feasible for settings with limited resources such as in clinical practice. In the within-subject comparison in SCD, each participant provides their own control data, thereby controlling for confounding variables, such as gender, age, socio-economic status, cognition, home environment, and parallel interventions (Lobo et al., 2017; Smith, 2012). Further, while randomization is certainly possible in SCDs, in contrast with RCTs. It is not a strict requirement do so. This makes it a suitable method for studies in which randomization is not feasible. In contrast to RCTs, SCD does not report data on group means, but determines whether interventions work on an individual level (Lobo et al., 2017). This allows researchers to examine for whom an intervention works and for whom it does not. And finally, a well-designed SCD is able to determine the probability of a causal relationship between the intervention and the outcomes and to generalize the findings to a broader population (Lobo et al., 2017).

RCTs often rely exclusively on nomothetic measures (i.e., standard questionnaires measuring one’s score on a predetermined dimension using predefined items that are consistent across all participants). SCD however allows for the use of idiographic outcome measures. Idiographic measures are standardized in design (e.g., 5 items rated on a 7-point Likert scale), but consist of participant generated items that therefore differ between participants. While nomothetic measures can contain items that are irrelevant to individual participants, idiographic outcome measures are able to examine change in complaints and goals most important to the individual participant. They are thereby more congruous with the reality of clinical settings (Lloyd et al., 2019; Sales & Alves, 2016). Also, idiographic items overcome some challenges nomothetic measures encounter. An example is the normative nature of preset questionnaires, while items can have different meanings to individual participants (Lloyd et al., 2019; Sales & Alves, 2016). All in all, SCD makes for a scientifically rigorous research design, well suited to be conducted in clinical settings.

The current study aims to further examine the effectiveness of MwyB/T on parental stress, parental self-efficacy, maternal internalizing problems and personalized items (consisting of individual treatment goals and clinical complaints). While the intervention was completed in small groups, in this study a SCD was used to assessed effects for individual cases. Our first aim was to determine common themes in these treatment goals and clinical complaints. Second, we aimed to examine the effectiveness of MwyB/T in improving outcomes on these common themes, using both idiographic and nomothetic data. Our third aim was assessing the effectiveness of the MwyB/T training on improving scores on nomothetic outcome measures regarding mindfulness, mindful parenting, parental self-efficacy, maternal internalizing problems, and parental stress. Our fourth aim was to assess to what extent improvement was dependent on the quantity of practice. Our fifth and final aim was to examine whether effectiveness of the MwyB/T training was dependent on training or group characteristics.

## Methods

### Design

The study has an ABCD design, with baseline phase (A), intervention phase (B), post-test phase (C), and follow-up phase (D). For every participant three to seven idiographic items were constructed before the start of the baseline phase. During all phases these questions were administered daily. At the start of the baseline phase, the post-test phase, and the follow-up phase nomothetic measures were administered once. Due to the heterogeneity of the target group, and ethical concerns about withholding care, allocation to control conditions (e.g. care-as-usual or waitlist control) was not feasible. Furthermore, due to the strict group intervention protocols, randomization in intervention characteristics such as duration was not possible either. And finally, randomization based on baseline length was not possible, as participants are often referred to the group training shortly before it starts.

The baseline phase functions as a personal criterion, to which scores from other phases can be compared (within subject comparison). The baseline phase, post-test phase, and follow-up phase were all designed to last 14 days. However, some participants were referred to the training very close to the starting date of the group training. Therefore, the baseline was shorter for these participants. For all participants there was a baseline of at least seven days. The intervention phase lasted 8 weeks for the mother-infant dyads, and 9 weeks for the mother-toddler dyads. The post-test phase took place directly after the intervention phase. The follow-up phase followed a single session booster, approximately 9 weeks after the last session of the intervention phase. The study was approved by the University of Amsterdam Ethics Review Board (2018-CDE-9039).

### Participants

The sample consisted of 10 mothers (age: *M* = 33.60 years, *SD* = 4.88) of infants (*n* = 5; age in months: *M* = 5.2, *SD* = 2.28) or toddlers (*n* = 5; age in months: *M* = 25.40; *SD* = 12.18). At the baseline measurement, mothers completed a questionnaire on sociodemographic characteristics. Four mothers (40%) reported they were working in a part-time (*n* = 3) or full-time (*n* = 1) job. The other six (60%) were either on maternity leave (*n* = 2), unemployed (*n* = 1), a student (*n* = 1), or a stay-at-home mother (*n* = 2). Eight mothers (80%) obtained a college degree or higher and two mothers completed secondary school. Four mothers (40%) had a solely Dutch cultural background, four (40%) had a mixed background, and 2 (20%) participants a non-Dutch background. Of both mothers and children 9 (90%) were white. Forty percent of the children were girls. Eight children lived in a two-parent household. Two mothers were in a co-parenting arrangement, with their children spending part of their time at their fathers’ home. Demographic information on all ten participating mothers and their children is depicted in Table 1.

**Table 1.** Demographics.

	Mother				Child		Family Situation	
ID	Age	Cultural Background	Education	Employment	Gender	Age in Months	Family Type	Siblings
1	35	Mixed	University degree	Maternity leave/38 h	Girl	3	Two-parent household	0
2	38	Non-Dutch	College degree	Maternity leave/32 h	Boy	5	Two-parent household	0
3	31	Dutch	College degree	40 h	Girl	16	Two-parent household	0
4	24	Mixed	Secondary education	Student	Girl	16	Co-parenting	0
5	36	Dutch	College degree	Unemployed	Boy	29	Two-parent household	0
6	38	Dutch	Secondary education	Stay-at-home mother	Boy	41	Co-parenting	1
7	38	Mixed	College degree	16 h	Boy	3	Two-parent household	0
8	33	Dutch	University degree	32 h	Boy	8	Two-parent household	0
9	27	Mixed	College degree	16 h	Boy	7	Two-parent household	0
10	36	Non-Dutch	University degree	Stay-at-home mother	Girl	45	Two-parent household	1

*Note.* Secondary education = senior general secondary education or pre-university education.

### Procedure

#### Participant Recruitment

MwyB/T is offered in (1) a clinical setting to mothers who are referred by their general practitioner and (2) in a non-clinical setting. During an intake at an outpatient treatment center located in the Netherlands, a psychologist (second author) and a mindfulness trainer assessed whether the MwyB/T training was a suitable treatment for the clinically referred mothers. In the non-clinical setting only a mindfulness trainer was present during this intake. Exclusion criteria for participation were postpartum psychoses, severe depression, severe post-traumatic stress disorder, and an inadequate proficiency in the Dutch or English language. Fourteen mothers referred to the training were approached for participation. Of them 11 mothers agreed on participating. One mother dropped out of the study but continued the training. All mothers participated voluntary and gave informed consent.

#### Intervention

MwyB/T (Potharst et al., 2017; Potharst, Zeegers, & Bögels, 2021) is a group training for mothers of young children (0–48 months) experiencing maternal mental health problems and/or stress or insecurities related to motherhood. The training consists of eight weekly two-hour sessions for the mothers with infants and nine weekly two-hour sessions for the mothers with toddlers. During part of the sessions the babies/toddlers are present, so mothers are able to put theory into practice during the training sessions. In between sessions mothers are encouraged to (1) meditate, (2) practice with informal mindfulness in daily life, (3) practice with mindfulness in their interactions with their children, and (4) read a chapter in a workbook. Audio files are available to guide them through their meditations. For more information on the intervention itself see Potharst et al., 2017, Potharst, Zeegers, & Bögels, 2021. Recommended group size is 4–6 mothers, however due to the COVID-19 pandemic it was not possible to organize face-to-face group meetings at the treatment center for part of the groups. Therefore, some mothers received an individual face-to-face training and some mothers partook in online group sessions using Zoom (see Table 2 for training characteristics). Group training session were led by two mindful parenting trainers or by a mindful parenting trainer and co-trained by a master student working as a clinical intern at the outpatient treatment center. Individual sessions were led by a mindful parenting trainer only.

**Table 2.** Training Characteristics.

ID	Baby/Toddler	Group Size	Online/Face to Face	Home practice^a^				Other Remarks
				Informal Mindfulness^b^	Mindful Parenting^b^	Meditation Days^c^	Meditation Minutes^d^	
				*M (SD)*	*M (SD)*	%	*M (SD)*	
1	Baby	2	Face to face	3.70 (1.28)	3.74 (1.22)	51.1	3.81 (4.66)	With partner^e^
2	Baby	1	Online	4.97 (1.10)	5.69 (0.92)	81.4	16.86 (12.14)	Not weekly
3	Toddler	3	Online					Non-clinical
4	Toddler	3	Online	4.32 (1.54)	3.59 (1.62)	73.0	18.30 (30.86)	
5	Toddler	3	Combination^f^	5.57 (1.16)	5.82 (1.09)	92.5	19.30 (16.69)	No booster
6	Toddler	3	Combination^f^	3.80 (1.03)	3.63 (1.04)	86.4	21.95 (13.23)	No booster
7	Baby	5	Online	4.00 (1.10)	7.00 (0.00)	30.6	7.08 (13.17)	
8	Baby	3	Online	3.33 (0.93)	3.10 (1.34)	93.7	17.98 (11.57)	Non-clinical
9	Baby	1	Online	3.88 (0.76)	3.82 (0.81)	55.4	7.57 (12.76)	
10	Toddler	5	Combination^g^	2.23 (.43)	2.16 (0.43)	97.7	5.14 (2.47)	With partner^e^

^a^Rated daily during the intervention phase.

^b^To what extent informal mindfulness/mindful parenting homework practices were incorporated (range 1 = not at all to 7 = a great deal).

^c^Percentage of days meditated.

^d^Daily average of minutes meditated.

^e^The partner of the participant was one of the other clients in the group.

^f^Seven sessions took place online, the final two at the treatment center.

^g^Five sessions took place online, and the four sessions in which the toddlers were present took place at the treatment center.

### Outcome Measures

#### Idiographic Measures

After the intake, every respondent had an extra meeting (either online or by phone) to construct their idiographic items. This extra meeting took place with a psychologist (second author) or master student working as a clinical intern at the outpatient treatment center (first author). Idiographic items were constructed either as problem-focused (higher scores reflecting a higher level of the problem that was covered by the specific item) or goal-focused (higher scores reflecting more goal achievement) and were rated on a 7-point Likert scale ranging from *strongly disagree* (1) to *strongly agree* (7). During the construction of the items, information from the intake was used as a guiding point of reference. However, participants decided what problems and goals they wanted to work on in the training and what was most important to them.

#### Nomothetic Measures

##### Mindfulness

The Dutch version of the Five Facet Mindfulness Questionnaire Short Form (FFMQ-SF; Bohlmeijer et al., 2011; De Bruin et al., 2012) was used to assess aspects of mindfulness. The FFMQ short form consists of 24 items which are scored on a 5-point Likert scale (ranging from 1 = *never or very rarely true* to 5 = *very often or always true*). The FFMQ distinguishes five facets of mindfulness: observing, describing, acting with awareness, non-judging of inner experience, and nonreactivity to inner experience. Example items are “I pay attention to physical experiences, such as the wind in my hair or sun on my face” (observing), “I’m good at finding words to describe my feelings” (describing), “I find myself doing things without being aware of what I’m doing” (acting with awareness [reverse coded]), “I tell myself I shouldn’t be feeling the way I’m feeling” (non-judging of inner experience [reverse coded]), and “I watch my feelings without getting carried away with them” (nonreactivity to inner experience). Total (subscale) scores are obtained by summing item scores and higher scores indicate a higher degree of mindfulness. Internal consistency, validity, and sensitivity of the original FFMQ-SF and the Dutch version of the full length FFMQ are good (Bohlmeijer et al., 2011; De Bruin et al., 2012). Subscale Cronbach’s alpha for the five facets range from .75 to .87 for the original FFMQ-SF (Bohlmeijer et al., 2011).

##### Mindful Parenting

The Dutch version of the Interpersonal Mindfulness in Parenting Scale (IM-P; De Bruin et al., 2014; Duncan, 2007) was used to assess mindful parenting. The IM-P consists of 29 items scored on a 5-point Likert scale (ranging from 1 = *never true* to 5 = *always true*). Item scores are summed to obtain a total score with higher scores indicating higher degrees of mindful parenting. The Dutch version of the IM-P distinguishes 6 subscales: listening with full attention, compassion for the child, non-judgmental acceptance of parental functioning, emotional nonreactivity in parenting, emotional awareness of the child, and emotional awareness of self. The wording of some items was slightly adjusted to fit the age of the children better. Three items (4, 8, and 28) were deleted from the questionnaire for mothers of infants as they were not applicable for them. Example items are: “I pay close attention to my child when we are spending time together” (listening with full attention), “When my child is going through a difficult time, I try to give him/her the nurturing and caring he/she needs” (compassion for the child), “When things I try to do as a parent do not work out, I can accept them and move on” (non-judgmental acceptance of parental functioning), “I often react to quickly to what my child says or does” (emotional nonreactivity in parenting), “It is hard for me to tell what my baby is feeling” (emotional awareness of the child), and “When I’m upset with my baby, I notice how I am feeling before I take action” (emotional awareness of self). Internal consistency of total scale of the Dutch version of the IM-P is good (*α* = .89). Internal consistencies of most subscales are satisfactory, although not for emotional awareness of self (De Bruin et al., 2014).

##### Parental Self-efficacy

Parental self-efficacy was assessed using the Dutch version of the Self-Efficacy in the Nurturing Role questionnaire (SENR; Pederson et al., 1989; Verhage et al., 2013). The SENR consist of 16 items scored on a 7-point Likert scale (ranging from 1 = *not at all representative of me* to 7 = *strongly representative of me*). All items are statements about feeling of confident in parenting, such as “I feel confident in my role as a parent” and “I can soothe my baby easily when he or she is crying or fussing”. Scores of all statements are summed to obtain a total score, with a higher score indicating higher parental self-efficacy. Test-retest reliability and internal consistency of the original SENR are moderate to high (Hsu & Sung, 2008; Pedersen et al., 1989). Cronbach’s alpha for the Dutch version is .86 (Verhage et al., 2013).

##### Maternal Internalizing Problems

The Patient Health Questionnaire-4 (PHQ-4; Löwe et al., 2010) was used to assess maternal internalizing problems. The PHQ-4 contains four items (e.g., “How often over the past two weeks did you experience feeling down, depressed or hopeless?” and “How often over the past two weeks did you experience not being able to stop or control worrying?”). Items are rated on a 4-point Likert scale, ranging from *not at all* (0) to *nearly every day* (3). A total score is obtained by summing all item scores, with higher scores indicating a higher degree of internalizing problems. Psychometric properties are satisfactory (*α = .82;* Löwe et al., 2010).

##### Parental Stress

The Parenting Stress Questionnaire (in Dutch: Opvoedingsbelasting Vragenlijst, OBVL; Vermulst et al., 2015) is a Dutch scale used to assess the extent to which parents experience stress in relation to the care for their child. It consists of 34 items scored on a 4-point Likert scale (ranging from *Not true* to *Very true*). Total as well as subscale scores are obtained by summing all (relevant) items. Higher scores indicate a higher level of stress. The OBVL distinguishes 5 subscales: parent-child relationship problems, parenting problems, depressive mood, parental role restriction, and physical health problems. Example items are: “I feel happy with my child” (parent-child relationship problems), “I am patient with my child” (parenting problems), “Sometimes I do not see the point of living” (depressive mood), “My child keeps me from other activities” (parental role restriction), and “I have an upset stomach” (physical health problems). The internal consistencies of the total scale (*α = .91) as well as the subscales (range from α = .77 to α = .87)* of the OBVL are satisfactory (Vermulst et al., 2015).

#### Quantity of Practice

During the intervention phase, the quantity of home practice was rated by the participants on a daily basis. They rated (1) to what extent they practiced with informal mindfulness in their daily life (on a 7-point Likert scale ranging from 1 = *not at all* to 7 = *a great deal*), (2) to what extent they practiced with mindfulness in interaction with their child (on a 7-point Likert scale ranging from 1 = *not at all* to 7 = *a great deal*), and (3) how many minutes they spent meditating.

### Analyses

The idiographic items were coded in order to define themes that were important to the participants (the first aim of the study). Two assessors separately analyzed all idiographic items and clustered them by common themes. Both assessors were (research) master students in child development/developmental psychology, who had sufficient knowledge of mindfulness, and worked as interns at an outpatient treatment center specialized in mindfulness, where they (co-)trained at least one mindfulness intervention. These assessors independently named all clusters and then discussed cluster names and categorization until agreement was reached. Once consensus was reached one new assessor (the second author) categorized all idiographic items into the different themes the previous assessors defined. Interrater reliability (Cohen’s kappa [κ]) was calculated between the final categorization of the first two assessors and that of the third assessor. Any disagreements on items between assessments were discussed among the assessors until consensus was reached.

To assess the effectiveness of MwyB/T on improving the scores on idiographic themes (the second aim of the study), the scores of each participant’s items were examined using both visual and quantitative analyses. Using visual analysis, trends per phase (β_0_ + m*t, where β_0_ is the intercept, *m* is the slope, and *t* is time), mean scores per phase, and differences between phases in mean scores were examined for every item. The directions of trendlines were determined visually, and proportion of positive, negative, and stable trendlines were calculated per theme per phase. Nonoverlap of all pairs (NAP)-scores were calculated to determine the effect size of changes on the idiographic items, using the Shine App for Single-Case Data Analysis (Shiny SCDA; De et al., 2020). This method is specifically designed for SCDs. NAP-scores indicate the proportion of similar scores between two phases, by comparing each datapoint from one phase to each datapoint from another phase. NAP-scores range from 0 to 1, with a score >.05 indicating an effect in the expected direction (0.50 < NAP ≤.65 weak effect; 0.66 ≤ NAP ≤.92 moderate effect; 0.93 ≤ NAP ≤1 strong effect). NAP-scores were calculated comparing the intervention phase, the posttest phase, and the follow-up phase to the baseline phase. Triangulation (the process of examining an outcome from several perspectives, using multiple types of data) was used to verify the findings on the idiographic items, by comparing these to results on validated nomothetic measures corresponding with the themes. Each idiographic theme found, was linked to a corresponding (sub)scale of the predetermined nomothetic measures by the first author, in consultation with the second author. Reliable change indices (RCI; Jacobson & Truax, 1991) were calculated for each theme for participants on the nomothetic (sub)scale corresponding with that particular theme. The RCI is a descriptive that indicates whether the difference between two phases is reliably greater than chance for an individual participant and controls for error. It is derived from the difference score between phases, and the Cronbach’s α and the standard deviation of the measure. RCIs have a normal distribution with a mean of zero and an SD of 1. An RCI of zero indicates no change in scores between phases, and an RCI of 1 indicates a difference equal to its standard error. RCIs of at least +1.64 or −1.64 indicate reliable change at *p* ≤ .05, and RCIs of at least +1.96 or −1.96 indicate reliable change at *p* ≤ .025. Where possible, psychometrics from the instruments’ manual were used for the RCI’s. If those were unavailable, psychometrics from validation studies were used.

For the third aim of the study, assessing effectiveness on the predetermined nomothetic measures, RCI’s (Jacobson & Truax, 1991) were calculated for all nomothetic instruments. Again, preferably psychometrics from the instruments’ manual, and otherwise from validation studies were used for the RCI’s.

Pearson correlations were calculated to assess the relationship between difference scores on all nomothetic outcome measures and practice quantity, the fourth aim of the study. If a Shapiro- Wilk test indicated a nonnormal distribution, Spearman correlations were used instead.

Our group is too small and heterogeneous to run reliable statistical tests based on personal or group characteristic differences. Therefore, for the fifth and final aim of the study, we tried to identify any remarkable differences in outcomes based on these characteristics by comparing results from all previous analyses.

## Results

### Aim 1: Defining Themes

A total of 63 items were constructed by the participants (*M* = 6.30, range 3–7). Two participants created both goal-oriented as well as problem-oriented items. All other participants only created goal-oriented items. Eleven themes were defined regarding different aspects of mindfulness, mindful parenting, parental self-efficacy, internalizing problems, self-care, and the parent-child relationship. Cohen’s kappa indicated a very good interrater reliability (κ = .91, 95% CI [.84, .98], *p* < .001).

Participant 1 created seven items, concerning the themes mindfulness: emotional non-reactivity, mindful parenting: attention, parental self-efficacy, internalizing problems (two items), and self-care (two items). The six items of participant 2 concerned mindfulness: awareness, mindfulness: emotional non-reactivity (two items), mindful parenting: attention, mindful parenting: awareness of the emotions of the child, and self-care. Participant 3 created six items concerning mindfulness: emotional non-reactivity, mindful parenting: emotional nonreactivity, parental self-efficacy (two items), self-care, and the parent-child relationship. Participant 4 created seven items concerning mindfulness: awareness (two items), mindfulness: emotional non-reactivity (two items), mindful parenting: attention, and internalizing problems (two items). The seven items participant 5 created concerned mindfulness: awareness (two items), mindfulness: emotional non-reactivity (two items), mindfulness: nonjudging of the inner experience, and self-care (two items). Participant 6 created seven items concerning mindfulness: emotional non-reactivity, mindful parenting: attention, mindful parenting: emotional awareness of the child, mindful parenting: emotional non-reactivity (2 items), and self-care (two items). Participant 7 created 6 items concerning mindful parenting: nonjudgmental acceptance of parental functioning, parental self-efficacy (two items), internalizing problems, self-care, and the parent-child relationship. Participant 8 created three items concerning mindfulness: awareness, mindful parenting: attention, and internalizing problems. Participant 9 created seven items, concerning mindfulness: emotional non-reactivity, mindful parenting: attention (two items), mindful parenting: emotional awareness of the child, mindful parenting: emotional non-reactivity (two items), and self-care. Participant 10 created seven items concerning mindful parenting attention (two items), mindful parenting: emotional nonreactivity, mindful parenting: nonjudgmental acceptance of parental functioning, parental self-efficacy, self-care, and the parent-child relationship.

After we defined themes, we determined a corresponding validated nomothetic (sub)scale to be able to triangulate our findings on the effectiveness of MwyB/T on the idiographic items. See Table 3 for the distribution of the idiographic items over the 11 themes, and their corresponding nomothetic subscales.

**Table 3.** Idiographic Themes and Their Corresponding Nomothetic (Sub)scales.

Theme	Example Item	Number of Items	Corresponding Nomothetic (Sub)scale
Mindfulness: Awareness	*Today I was able to keep my attention in the here-and-now.*	6	FFMQ-SF acting with awareness
Mindfulness: Emotional nonreactivity	*Today I was able to remain calm during stressful moments.*	10	FFMQ-SF nonreactivity to inner experience
Mindfulness: Nonjudging of inner experience	*Today I could accept that not everything is positive or can be solved.*	1	FFMQ-SF nonjudging of inner experience
Mindful parenting: Attention	*Today I breastfed my child with full attention.*	9	IM-P listening with full attention
Mindful parenting: Emotional awareness of the child	*Today I was able to empathize with my child’s experiences.*	3	IM-P emotional awareness of the child
Mindful parenting: Emotional nonreactivity in parenting	*Today I was able to make conscious decisions in how to react to my child’s emotions.*	6	IM-P emotional nonreactivity in parenting
Mindful parenting: Nonjudgmental acceptance of parental functioning	*I could accept that I am not always able to help my children.*	2	IM-P nonjudgmental acceptance of parental functioning
Parental self-efficacy	*Today I felt I am a good mother.*	6	SENR
Internalizing problems mother	*Today I was happy. I did not feel sad.*	6	PHQ4
Self-care	*Today I took the time to do nice things for myself.*	11	OBVL parental role restriction
Parent-child relationship	*Today I felt happy with my child.*	3	OBVL parent-child relationship problems

*Note.* Example item = an example of an idiographic item constructed by the participants, categorized to this specific theme. FFMQ-SF = Five Facet Mindfulness Questionnaire-Short Form. IM-P = Interpersonal Mindfulness in Parenting Scale. SENR = Self-Efficacy in the Nurturing Role questionnaire. PHQ-4 = Patient Health Questionnaire-4. OBVL = Parental Stress Questionnaire.

### Aim 2: Effectiveness MwyB/T on Idiographic Themes

#### Visual Analysis

All mothers completed the intervention and therefor data were available for all mothers. However, for Participant 2, no idiographic data were available for the intervention and post-test phase. For participant 3 and participant 10 no idiographic data were available for the post-test phase. For ease of interpretation problem-oriented items were recoded, so an increase in scores depicts a change in the expected direction for all items.

**Figure 1.**
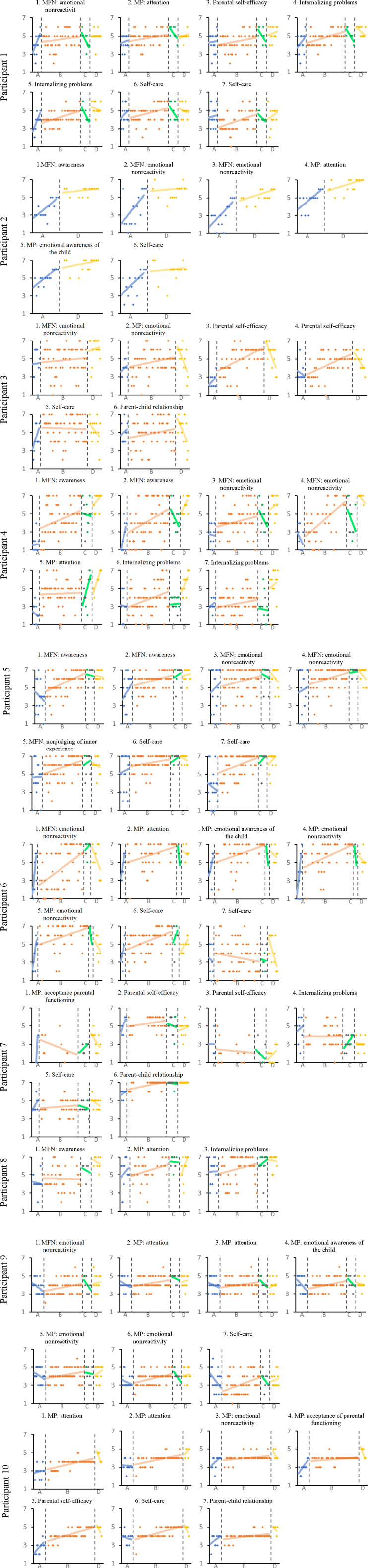
Graphs of the Scores of the Daily Idiographic Items. *Note.* MFN = Mindfulness. MP = Mindful Parenting. A = Baseline Phase. B = Intervention Phase. C = Posttest Phase. D = Follow-Up Phase.

See Figure 1 for graphs including trendlines of the daily scores of the idiographic items. In Table 4 the direction of the slopes of the trendlines is summarized. While it was expected that most baselines would indicate a deteriorating or stable phase, for 41 out of 63 items (65%) the slope of the baseline trendline already indicates improvement during the phase. As expected, intervention phase trendline slopes were positive in 49 of the 57 items (86%; for six items no intervention phase data were available). This means there was an increased improvement in scores during the intervention period. Trendline slopes we positive in 10 out of 44 items (23%; idiographic post-test data were missing for participants 2, 3, and 10), and negative in 28 out of the items (64%). And finally, trend line slopes for the follow-up phase were positive in 28 out of 63 items (44%), and negative in 30 out of the items (48%). For percentages of slopes positive, negative, and stable per phase per theme, see Table 4.

**Table 4.** Directions of the Trend Line Slopes for all Idiographic Items and Percentages of Item Slopes Positive, Negative, and Stable per Theme.

ID	Mindfulness																Mindful Parenting												Parental Self-Efficacy				Internalizing Problems				Self-care				Parent-child Relationship			
	Awareness				Emotional Nonreactivity				Nonjudging of Inner Experience				Attention				Emotional Awareness of the Child				Emotional Nonreactivity in Parenting				Nonjudgmental Acceptance of Parental Functioning																			
	A	B	C	D	A	B	C	D	A	B	C	D	A	B	C	D	A	B	C	D	A	B	C	D	A	B	C	D	A	B	C	D	A	B	C	D	A	B	C	D	A	B	C	D
1					+	+	−	o					+	+	−	+													+	+	−	+	+	+	−	−	+	+	−	−				
																																	+	+	−	−	+	+	−	+				
2	+			+	+			+					+			+	+			+																	+			+				
					+			+																																				
3					+	+		+													+	+		−					+	+		−					+	o		−	+	+		−
																													−	+		−												
4	+	+	−	+	−	+	−	+					−	+	+	+																	−	+	+	+								
	+	+	−	+	−	+	−	−																									+	+	−	+								
5	−	+	−	−	+	+	−	−	o	+	+	−																									+	+	+	−				
	+	+	+	+	+	+	+	−																													−	+	+	+				
6					+	+	+	−					+	+	−	−	+	+	−	−	+	+	−	−													+	+	+	+				
																					+	+	−	−													−	−	−	−				
7																									+	−	+	−	+	+	−	+	+	o	+	o	+	+	−	−	+	+	−	o
																													o	−	−	+												
8	−	o	−	−									+	+	o	+																	o	+	+	−								
9					−	+	−	+					−	+	−	+	−	+	−	−	−	+	−	+													−	+	−	+				
													−	+	−	+					−	+	−	+																				
10													+	+		−					+	+		−	+	+		−	+	+		−					−	+		−	+	+		−
													o	+		+																												
% +	67	80	20	67	70	100	29	50	0	100	100	0	55	100	17	78	67	100	0	33	67	100	0	33	100	50	100	0	67	83	0	50	67	83	50	33	64	80	38	45	100	100	0	0
% −	33	0	80	33	30	0	71	40	0	0	0	100	33	0	67	22	0	0	100	67	33	0	100	67	0	50	0	100	17	17	100	50	17	0	50	50	36	10	62	55	0	0	100	67
% o	0	20	0	0	0	0	0	10	100	0	0	0	11	0	17	0	33	0	0	0	0	0	0	0	0	0	0	0	17	0	0	0	17	17	0	17	0	10	0	0	0	0	0	33

*Note.* ID = participant identification number. A = baseline phase. B = intervention phase. C = post-test phase. D = follow-up phase. % + = the percentage of trendline slopes that are positive. % − = the percentage of trendline slopes that are negative. % o = the percentage of trendline slopes that are stable.

+ indicates improvement. − indicates deterioration. o indicates stability.

In Table 5 mean scores for all items for all participants are depicted per phase, as well as difference scores between the phases. For most items (54%), there was a continuous improvement in mean scores over time at all phases. While we found some deterioration (0.1–1.6) on items between different phases, comparing the intervention phase means to the baseline means we found improvement (0.1–2.8) on 84% of the items (*n* = 57). Comparing post-test phase means to baseline means we found improvement (0.1–3.3) on 86% of the items (*n* = 44). Comparing follow-up scores to baseline scores (*n* = 63), only participant 7’s items 2, 3, and 4, and participant 9’s item 3 show a deterioration (0.2, 1.3, 1.6, and 0.1 respectively). All other scores (94%) indicate an improvement between the baseline and the follow-up phase (0.1–4.9).

**Table 5.** Mean Scores of the Idiographic Items for all Participants, and the Difference Scores Between Phases.

Item		Participant 1				Participant 2				Participant 3				Participant 4				Participant 5			
		Phase				Phase				Phase				Phase				Phase			
		A	B	C	D	A	B	C	D	A	B	C	D	A	B	C	D	A	B	C	D
1	Mean	4.2	4.6	4.6	5.0	3.7	–	–	5.7	4.4	4.8	–	6.1	1.6	4.4	4.9	6.5	4.1	5.6	6.4	6.3
	Dif. w/A		0.4	0.4	0.8		–	–	2.0		0.4	–	1.7		2.8	3.3	4.9		1.5	2.3	2.2
	Dif. w/B			0.0	0.4			–	–			–	1.3			0.5	2.1			0.8	0.7
	Dif. w/C				0.4				–				–				1.6				−0.1
2	Mean	4.4	4.8	5.4	5.0	3.6	–	–	5.6	3.9	4.6	–	5.0	2.3	4.2	4.6	6.0	4.6	5.8	6.4	6.4
	Dif. w/A		0.4	1.0	0.6		–	–	2.0		0.7	–	1.1		1.9	2.3	3.7		1.2	1.8	1.8
	Dif. w/B			0.6	0.2			–	–			–	0.4			0.4	1.8			0.6	0.6
	Dif. w/C				−0.4				–				–				1.4				0.0
3	Mean	4.4	4.4	5.1	5.3	3.1	–	–	5.2	2.6	4.9	–	5.5	2.7	4.2	4.5	5.9	5.0	5.7	6.3	6.1
	Dif. w/A		0.0	0.7	0.9		–	–	2.1		2.3	–	2.9		1.5	1.8	2.2		0.7	1.3	1.1
	Dif. w/B			0.7	0.9			–	–			–	0.6			0.3	1.7			0.6	0.4
	Dif. w/C				0.2				–				–				1.2				−0.2
4	Mean	4.3	4.8	5.1	5.5	4.8	–	–	6.4	3.4	4.4	–	4.9	2.3	4.1	4.6	6.9	4.8	6.0	6.8	6.4
	Dif. w/A		0.5	0.8	1.2		–	–	1.6		1.0	–	1.5		1.8	2.6	4.6		1.2	2.0	1.6
	Dif. w/B			0.3	0.7			–	–			–	0.5			0.5	2.8			0.8	0.4
	Dif. w/C				0.4				–				–				2.3				−0.4
5	Mean	3.7	4.2	4.6	5.2	5.0	–	–	6.5	4.3	5.5	–	5.3	2.2	4.4	4.8	6.1	4.7	5.7	6.3	6.0
	Dif. w/A		0.5	0.9	1.5		–	–	1.5		1.2	–	1.0		2.2	2.6	3.9		1.0	1.6	1.3
	Dif. w/B			0.4	1.0			–	–			–	−0.2			0.4	1.7			0.6	0.3
	Dif. w/C				0.6				–				–				1.3				−0.3
6	Mean	4.6	4.7	4.9	5.0	4.4	–	–	5.8	5.0	4.9	–	5.3	3.1	3.8	3.3	5.6	5.3	6.2	6.6	6.7
	Dif. w/A		0.1	0.3	0.4		–	–	1.4		−0.1	–	0.3		0.7	0.2	2.5		0.9	1.3	1.4
	Dif. w/B			0.2	0.3			–	–			–	0.4			−0.5	1.8			0.4	0.5
	Dif. w/C				0.1				–				–				2.3				0.1
7	Mean	4.4	3.8	4.3	4.4									3.2	3.3	2.7	5.4	3.6	6.0	6.7	6.5
	Dif. w/A		−0.6	−0.1	0.0										0.1	−0.5	2.2		2.4	3.1	2.9
	Dif. w/B			0.5	0.6											−0.6	2.1			0.7	0.5
	Dif. w/C				0.1												2.7				−0.2
		Participant 6				Participant 7				Participant 8				Participant 9				Participant 10			
		Phase				Phase				Phase				Phase				Phase			
	Phase	A	B	C	D	A	B	C	D	A	B	C	D	A	B	C	D	A	B	C	D
1	Mean	3.6	4.3	6.6	5.3	2.5	2.9	2.7	3.5	4.2	4.6	5.6	6.4	3.8	3.7	4.1	4.2	2.9	3.8	−	4.6
	Dif. w/A		0.7	3.0	1.7		0.4	0.2	1.0		0.4	1.4	2.2		−0.1	0.3	0.4		0.9	−	1.7
	Dif. w/B			2.3	1.0			−0.2	0.6			1.0	1.8			0.4	0.5			−	0.8
	Dif. w/C				−1.3				0.8				0.8				0.1				−
2	Mean	4.6	5.9	5.8	6.0	4.9	5.3	5.1	4.7	5.2	5.4	6.4	5.9	4.1	4.2	4.8	4.4	3.2	3.9	−	4.6
	Dif. w/A		1.3	1.2	1.4		0.4	0.2	−0.2		0.2	1.2	0.7		0.1	0.7	0.3		0.7	−	1.4
	Dif. w/B			−0.1	0.1			−0.2	−0.6			1.0	0.5			0.6	0.2			−	0.7
	Dif. w/C				0.2				−0.4				−0.5				−0.4				−
3	Mean	4.6	6.0	5.8	5.9	3.0	2.3	1.9	1.7	5.4	5.7	6.3	6.6	4.0	4.0	4.2	3.9	3.5	4.0	−	4.4
	Dif. w/A		1.4	1.2	1.3		−0.7	−1.1	−1.3		0.3	0.9	1.2		0.0	0.2	−0.1		0.5	−	0.9
	Dif. w/B			−0.2	−0.1			−0.4	−0.6			0.6	0.9			0.2	−0.1			−	0.4
	Dif. w/C				0.1				−0.2				0.3				−0.3				−
4	Mean	3.7	5.7	5.8	5.3	4.7	3.9	3.3	3.1					4.0	4.0	4.3	4.1	3.0	3.9	−	4.6
	Dif. w/A		2.0	2.1	1.6		−0.8	−1.4	−1.6						0.0	0.3	0.1		0.9	−	1.6
	Dif. w/B			0.1	−0.4			−0.6	−0.8							0.3	0.1			−	0.7
	Dif. w/C				−0.5				−0.2								−0.2				−
		Participant 6				Participant 7				Participant 8				Participant 9				Participant 10			
		Phase				Phase				Phase				Phase				Phase			
	Phase	A	B	C	D	A	B	C	D	A	B	C	D	A	B	C	D	A	B	C	D
5	Mean	3.9	5.9	6.0	4.7	4.4	4.3	4.3	4.8					4.0	4.1	4.3	4.4	2.7	4.3	−	4.8
	Dif. w/A		2.0	2.1	0.8		−0.1	−0.1	0.4						0.1	0.3	0.4		1.6	−	2.1
	Dif. w/B			0.1	−1.2			0.0	0.5							0.2	0.3			−	0.5
	Dif. w/C				−1.3				0.5								0.1				−
6	Mean	4.0	5.4	5.9	4.9	5.8	6.8	6.9	7.0					3.3	3.7	4.0	4.1	3.8	4.4	−	4.8
	Dif. w/A		1.4	1.9	0.9		1.0	1.1	1.2						0.4	0.7	0.8		0.6	−	1.0
	Dif. w/B			0.5	−0.5			0.1	0.2							0.3	0.4			−	0.4
	Dif. w/C				−0.5				0.1								0.1				−
7	Mean	3.3	3.5	3.3	4.2									3.5	3.1	3.6	3.6	3.5	4.0	−	4.8
	Dif. w/A		0.2	0.0	0.9										−0.4	0.1	0.1		0.5	−	1.3
	Dif. w/B			−0.2	0.7											0.5	0.5			−	0.8
	Dif. w/C				0.9												0.0				−

*Note.* A = baseline phase. B = intervention phase. C = post-test phase. D = follow-up phase. Dif. w/= difference with.

Positive difference scores indicate improvement over time. Negative difference scores indicate deterioration over time.

Table 6 depicts mean scores per phase for all themes, as well as difference scores between phases. Again we found some deterioration comparing post-test means to intervention means (27%; 0.2–0.7) and comparing follow-up to post-test means (0.1–1.2) later phases (36%; 0.1–1.2). However, comparing mean scores during the intervention phase, the post-test phase, and the follow-up phase to baseline phase scores, difference scores indicated improvement on all themes (100%, 0.9–2.8).

**Table 6.** Mean Scores of the Idiographic Items for all Participants, and the Difference Scores Between Phases.

Theme	Phase	*n*	Mean	Dif. w/A	Dif. w/B	Dif. w/C	Theme	Phase	*n*	Mean	Dif. w/A	Dif. w/B	Dif. w/C
MFN: Awareness	A	6	3.4				MP: Nonjudgmental acceptance of parental functioning	A	2	2.8			
	B	5	4.9	1.5				B	2	3.4	0.7		
	C	5	5.6	2.2	0.7			C	1	2.7	0.0	−0.7	
	D	6	6.2	2.8	1.3	0.6		D	2	4.1	1.3	0.7	1.4
MFN: Emotional nonreactivity	A	10	3.8				Self-efficacy	A	6	3.5			
	B	8	4.7	0.9				B	6	4.3	0.8		
	C	7	5.4	1.6	0.7			C	3	4.0	0.5	−0.2	
	D	10	5.7	1.9	1.0	0.3		D	6	4.5	1.0	0.2	0.5
MFN: Nonjudgement of inner experience	A	1	4.7				Internalizing problems	A	6	4.1			
	B	1	5.7	1.0				B	6	4.4	0.3		
	C	1	6.3	1.6	0.6			C	6	4.2	0.2	−0.2	
	D	1	6.0	1.3	0.3	−0.3		D	6	5.2	1.2	0.9	1.0
MP: Attention	A	9	3.9				Self-care	A	11	4.1			
	B	8	4.6	0.6				B	10	4.7	0.5		
	C	6	5.2	1.3	0.7			C	8	5.0	0.8	0.3	
	D	9	5.1	1.2	0.6	−0.1		D	11	5.1	0.9	0.4	0.1
MP: Emotional awareness of the child	A	3	4.5				Parent-child relationship	A	3	4.8			
	B	2	5.0	0.5				B	3	5.2	0.5		
	C	2	5.1	0.5	0.0			C	1	6.9	2.1	1.7	
	D	3	5.5	1.0	0.5	0.5		D	3	5.7	0.9	0.5	−1.2
MP: Emotional nonreactivity in parenting	A	6	3.7										
	B	6	4.7	1.0									
	C	4	5.0	1.3	0.4								
	D	6	4.7	0.9	0.0	−0.4							

*Note. n* = number of items included. A = baseline phase. B = intervention phase. C = post-test phase. D = follow-up phase. Dif. W/= difference with. Positive difference scores indicate improvement over time. Negative difference scores indicate deterioration over time.

The first three themes deduced from the idiographic items related to the broader construct mindfulness, namely mindfulness: awareness, mindfulness: emotional nonreactivity, and mindfulness: nonjudging of inner experience. All four mothers who created items concerning the theme mindfulness: awareness showed improvement on this theme, on all their idiographic items (most a moderate effect during intervention and at posttest, and all a strong effect at follow-up; see Table 7 for all idiographic results per participant). In congruence with this, all four mothers improved on the corresponding nomothetic subscale (see Table 8 for all nomothetic results per participant). Seven of the participants created one or more items concerning the theme mindfulness: emotional nonreactivity. All of them showed improvement in their emotional nonreactivity (weak to moderate effects during the intervention phase, and weak to strong effects at posttest and follow-up), although results between idiographic and nomothetic measures were not congruent for all. One mother created an item concerning mindfulness: nonjudging of inner experience. Scores on her idiographic item (moderate effect) as well as the corresponding subscale showed improvement.

**Table 7.** NAP Scores Comparing Idiographic Scores During the Intervention Phase, Posttest Phase, and Follow-Up Phase to the Baseline Phase.

ID	Mindfulness									Mindful Parenting											
	Awareness			Emotional Nonreactivity			Nonjudging of Inner Experience			Attention			Emotional Awareness of the Child			Emotional Nonreactivity in Parenting			Nonjudgmental Acceptance of Parental Functioning		
	AB	AC	AD	AB	AC	AD	AB	AC	AD	AB	AC	AD	AB	AC	AD	AB	AC	AD	AB	AC	AD
1				*0.61*	*0.61*	***0.74***				0.36	0.47	*0.54*									
2			**0.96**			***0.79***						***0.90***			***0.89***						
						***0.91***															
3				*0.55*		***0.80***										***0.71***		***0.77***			
4	***0.91***	**0.97**	**1**	***0.79***	***0.85***	**0.96**				***0.90***	***0.91***	**1**									
	***0.77***	***0.85***	**0.98**	***0.76***	***0.82***	**1**															
5	***0.77***	***0.90***	***0.88***	*0.55*	***0.66***	*0.61*	***0.70***	***0.78***	***0.75***												
	***0.68***	***0.82***	***0.82***	*0.63*	***0.74***	***0.69***															
6				*0.60*	**0.94**	***0.76***				***0.79***	***0.79***	***0.84***	***0.80***	***0.75***	***0.78***	***0.77***	***0.79***	***0.73***			
																***0.84***	***0.89***	*0.65*			
7																			*0.60*	*0.50*	***0.68***
8	*0.59*	***0.85***	**0.97**							*0.51*	***0.75***	*0.64*									
9				0.47	*0.61*	*0.62*				*0.54*	***0.79***	*0.64*	0.48	*0.59*	*0.53*	*0.52*	*0.60*	*0.61*			
										*0.50*	0.58	0.47				***0.66***	***0.75***	***0.75***			
10										***0.82***		**0.96**				***0.68***		***0.81***	***0.86***		**0.96**
										***0.78***		**0.95**									

ID	Parental Self-Efficacy			Internalizing Problems			Self-care			Parent-child Relationship		
	AB	AC	AD	AB	AC	AD	AB	AC	AD	AB	AC	AD
1	*0.63*	***0.82***	***0.74***	0.48	***0.70***	***0.75***	*0.64*	***0.75***	***0.90***			
				*0.62*	***0.73***	***0.84***	*0.54*	*0.58*	**0.63**			
2									***0.81***			
3	***0.91***		**0.96**				***0.72***		***0.70***	*0.50*		*0.59*
	***0.73***		***0.85***									
4				*0.63*	*0.56*	**0.93**						
				*0.54*	0.35	***0.88***						
5							*0.60*	***0.67***	***0.70***			
							***0.83***	***0.89***	***0.88***			
6							***0.78***	***0.84***	***0.71***			
							*0.53*	*0.50*	***0.67***			
7	***0.66***	*0.58*	0.47	***0.67***	***0.80***	***0.83***	0.48	0.47	***0.66***	***0.90***	**0.97**	**1**
	0.35	0.24	0.24									
8				*0.58*	***0.72***	***0.78***						
9							0.39	*0.53*	*0.53*			
10	**0.94**		**0.99**				***0.74***		***0.88***	***0.66***		**0.94**

*Note.* ID = participant identification number. A = baseline phase. B = intervention phase. C = posttest phase. D = follow-up phase.

NAP <.50 effect opposite of the expected direction. NAP >.50 effect in the expected direction.

0.50 < NAP ≤.65 weak effect (cursive). 0.66 ≤ NAP ≤.92 moderate effect (cursive and bold). 0.93 ≤ NAP ≤1 strong effect (bold).

**Table 8.** RCIs Nomothetic (Sub)scales Corresponding to Participants’ Idiographic Items.

	FFMQ						IM-P								SENR		PHQ4		OBVL			
ID	Acting with Awareness		Nonreactivity to Inner Experience		Nonjudging of Inner Experience		Listening with Full Attention		Emotional Nonreactivity in Parenting		Emotional Awareness of the Child		Nonjudgmental Acceptance of Parental Functioning						Parental Role Restriction		Parent-Child Relationship Problems	
	AC	AD	AC	AD	AC	AD	AC	AD	AC	AD	AC	AD	AC	AD	AC	AD	AC	AD	AC	AD	AC	AD
1			0.00	0.46			0.00	**3.80**								*−1.78*	**2.43**	**5.66**	−0.55	0.00		
2	**2.58**	**2.58**	**3.23**	**4.15**			**2.53**	**2.53**			**2.45**	**3.26**							***1.64***	1.09		
3			0.92	0.46					0.00	1.48					−1.43	0.00			*−1.64*	−1.09	0.00	0.78
4	**3.10**	**3.10**	**4.15**	**5.99**			**2.53**	**3.16**									0.81	**6.47**				
5	**2.58**	**5.17**	**3.23**	**5.53**	***1.89***	**5.20**													0.00	0.00		
6			1.38	−0.46			***1.90***	1.27	0.00	−1.48	1.63	0.82							***1.64***	*−3.28*		
7													0.76	0.38		−0.18	−1.62	−0.81	*−2.73*	*−1.64*	0.78	0.00
8	**2.58**	**3.10**					***1.90***	**4.43**									0.00	0.81				
9			1.38	***1.84***			−0.63	0.00	0.00	0.00	−0.82	0.00							1.09	0.55		
10								**2.53**		0.99				**3.79**		**1.96**				1.09		1.55

*Note.* ID = participant identification number. FFMQ-SF = Five Facet Mindfulness Questionnaire-Short Form. IM-P = Interpersonal Mindfulness in Parenting Scale. SENR = Self-Efficacy in the Nurturing Role questionnaire. PHQ-4 = Patient Health Questionnaire-4. OBVL = Parental Stress Questionnaire. PT = at posttest. FU = at follow-up.

Reliable deteriorations *p* ≤ .050 are cursive. Reliable improvements *p* ≤ .050 are cursive and bold. Reliable improvements *p* ≤ .025 are bold.

The following four themes were related to mindful parenting: mindful parenting: attention, mindful parenting: emotional awareness of the child, mindful parenting: emotional non-reactivity in parenting, and mindful parenting: nonjudgmental acceptance of parental functioning. Improvement on mindful parenting: attention was found for all seven mothers who created an item concerning this theme on their idiographic items, although for one mother there was only a weak effect at follow-up. For another mother there only was a weak effect at posttest that disappeared at follow-up for one of her items, but she showed a weak to moderate improvement on her other idiographic item. This was also the only mother that did not show improvement on the corresponding nomothetic subscale. One mother showed a weak to moderate improvement on her idiographic item, one showed a moderate effect, one a moderate to strong effect, and two mothers showed a strong effect at all phases. Three participants created an item concerning mindful parenting: emotional awareness of the child. Mother 9 only showed a weak effect on her idiographic item, mother 7 only showed a moderate effect on her idiographic item, and mother 2 showed moderate improvement on het idiographic item as well as reliable improvement on the nomothetic subscale. Of the four participants who created items regarding mindful parenting: emotional nonreactivity in parenting, none showed reliable change on the nomothetic subscale. However, while for one mother the effect remained weak, idiographic results for all participants did indicate improvement (moderate effect for three of the four mothers). Of the two participants who created an item regarding mindful parenting: nonjudgmental acceptance of parental functioning, one only showed a weak to moderate improvement on the idiographic subscale, while the other showed moderate to strong improvement on the idiographic item and reliable improvement on the nomothetic subscale.

Of the four mothers who created items regarding parental self-efficacy, one mother showed weak to moderate improvement on her idiographic item, but a deterioration at follow-up on the corresponding nomothetic subscale. One mother appeared to deteriorate on part of her idiographic items, but showed no change on the nomothetic scale. One mother showed moderate to strong improvement on her idiographic items, but not on the nomothetic scale. And one mother showed strong improvement on her idiographic item and reliable improvement on the nomothetic scale.

Of the four mothers who created items concerning internalizing problems, all showed improvement on their idiographic items, although for two mothers this was not the case for all their items during all phases. At follow-up all four mothers showed moderate to large improvement. For two mothers the improvement was also confirmed on the nomothetic scale.

Of the eight mothers creating items in the theme self-care, all showed improved scores on their idiographic items, although for one mother it remained weak. All 7 other mothers showed a moderate effect at follow-up. Only for participant 2 and 6 this improvement was corroborated by the nomothetic results, and only at posttest.

Three mothers created items about the parent-child relationship. One of them showed a weak improvement on her idiographic item, the other two showed a moderate to strong improvement. No changes were found on the nomothetic subscale.

For most items a growth in effect size was found throughout the phases.

### Aim 3: Effectiveness MwyB/T on Predetermined Outcomes

Table 9 presents the percentage of participants who experienced a reliable improvement in mindfulness, mindful parenting, parental self-efficacy, maternal internalizing problems, and parental stress, from baseline assessment to posttest and follow-up assessment. As psychometric information on the full scale of the FFMQ-SF was not available, we were unable to determine the RCI. Therefore, results on all subscales are depicted.

**Table 9.** Reliable Changes in Mindfulness, Mindful Parenting, Parental Stress, Parental Self-Efficacy, and Maternal Internalizing Problems From Baseline Assessment to Posttest and Follow-Up Assessment.

Outcome	Measure	(Sub)scale	Reliable Improvement at Posttest (%)	Reliable Improvement at Follow-up (%)	Reliable Improvement at Any Point (%)	Reliable Deterioration at Posttest (%)	Reliable Deterioration at Follow-up (%)	Reliable Deterioration at Any Point (%)
Mindfulness	FFMQ-SF	Observe	0/9, 0.0	0/10, 0.0	0/10, 0.0	0/9, 0.0	0/10, 0.0	0/10, 0.0
		Describe	0/9, 0.0	4/10, 40.0	4/10, 40.0	0/9, 0.0	0/10, 0.0	0/10, 0.0
		Acting with awareness	5/9, 55.6	6/10, 60.0	7/10, 70.0	0/9, 0.0	0/10, 0.0	0/10, 0.0
		Nonjudging of inner experience	4/9, 44.4	4/10, 40.0	5/10, 50.0	0/9, 0.0	1/10, 10.0	1/10, 10.0
		Nonreactivity to inner experience	3/9, 33.3	6/10, 60.0	6/10, 60.0	0/9, 0.0	0/10, 0.0	0/10, 0.0
Mindful parenting	IM-P	Total	3/9, 33.3	6/10, 60.0	6/10, 60.0	0/9, 0.0	0/10, 0.0	0/10, 0.0
		Attention	5/9, 55.6	6/10, 60.0	8/10, 80.0	0/9, 0.0	0/10, 0.0	0/10, 0.0
		Compassion for the child	2/9, 22.2	1/10, 10.0	1/10, 10.0	2/9, 22.5	3/10, 30.0	3/10, 30.0
		Nonjudgemental acceptance of parental functioning	3/9, 33.3	4/10, 40.0	5/10, 50.0	0/9, 0.0	0/10, 0.0	0/10, 0.0
		Emotional nonreactivity in parenting	2/9, 22.2	2/10, 20.0	3/10, 30.0	0/9, 0.0	0/10, 0.0	0/10, 0.0
		Emotional awareness of the child	1/9, 11.1	2/10, 20.0	2/10, 20.0	0/9, 0.0	0/10, 0.0	0/10, 0.0
		Emotional awareness of the self	1/9, 11.1	3/10, 30.0	3/10, 30.0	0/9, 0.0	0/10, 0.0	0/10, 0.0
Parental self-efficacy	SENR	Total	1/5, 20.0	2/8, 25.0	3/8, 37.5	1/5, 20.0	1/8, 12.5	2/8, 25.0
Maternal internalizing problems	PHQ-4	Total	2/9, 22.2	5/10, 50.0	5/10, 50.0	0/9, 0.0	0/10, 0.0	0/10, 0.0
Parental stress	OBVL	Total	5/9, 55.6	5/10, 50.0	6/10, 60.0	1/9, 11.1	1/10, 10	1/10, 10

*Note.* FFMQ-SF = Five Facet Mindfulness Questionnaire-Short Form. IM-P = Interpersonal Mindfulness in Parenting scale. SENR = Self-Efficacy in the Nurturing Role questionnaire. PHQ-4 = Patient Health Questionnaire-4. OBVL = Parental Stress Questionnaire.

### Aim 4: Effectiveness MwyB/T Dependent on Practice Quantity

Table 10 presents the correlations between the pretest to posttest difference scores on the abovementioned nomothetic outcomes and the practice quantity.

**Table 10.** Correlations Between Practice Quantity and Pretest-Posttest Difference Scores

Measure	(Sub)scale	Informal Mindfulness	Mindful Parenting	% Days Meditated	*M* Minutes Meditated
FFMQ-SF	Observe	.19	.44	.74*	.42
	Describe^a^	.30	.28	.33	.86
	Acting with awareness	.10	−.50	.74*	.69†
	Nonjudging of inner experience	.12	−.54	.77*	.69†
	Nonreactivity to inner experience	.61	−.16	.68†	.69†
IM-P	Total^a^	.62	.05	.71*	.64†
	Listening with full attention	.62	.27	.66†	.82*
	Compassion for the child	.71*	.54	.57	.70†
	Nonjudgmental acceptance of parental functioning	.58	.28	.74*	.63†
	Emotional nonreactivity in parenting	.60	−.01	.69†	.55
	Emotional awareness of the child	−.25	.18	−.30	−.17
	Emotional awareness of the self	.75*	.41	.06	.11
SENR	Total	.61	.62	−.09	.77
PHQ-4	Total	.31	−.34	.38	.11
OBVL	Total	.35	−.21	.59	.64†

*Note.* FFMQ-SF = Five Facet Mindfulness Questionnaire-Short Form. IM-P = Interpersonal Mindfulness in Parenting scale. SENR = Self-Efficacy in the Nurturing Role questionnaire. PHQ-4 = Patient Health Questionnaire-4. OBVL = Parental Stress Questionnaire.

*p ≤ .050, ^†^*p* ≤ .100.

^a^Spearman.

### Aim 5: Effectiveness MwyB/T Dependent on Training and Group Characteristics

Some mothers showed more improvement than others. However, when categorizing mothers by training or group characteristics (mothers of babies vs. mothers of toddlers, online vs. offline, groups vs. individual sessions, with vs. without partner, and a preventative vs. clinical setting), no clear differences were found in effectiveness of the intervention, based on these characteristics.

## Discussion

Earlier research on the effectiveness of MwyB/T, a training for parents of young children experiencing parental mental health problems and/or parenting related stress or insecurities, had methodological limitations. Non-randomized, and mostly quasi-experimental designs were used. The current study aimed to assess the effectiveness of MwyB/T in a more rigorous way, by making use of SCD. Aims were to (1) determine common themes in complaints and goals set by the participants, (2) assess the effectiveness of MwyB/T in improving outcomes on these themes, using both idiographic and nomothetic data, (3) to assess the effectiveness of the MwyB/T training on improving scores on predetermined nomothetic outcome measures regarding mindfulness, mindful parenting, parental self-efficacy, maternal internalizing problems, and parental stress, and (4) to what extent improvement is associated with home practice, and, finally, (5) to examine whether effectiveness of the MwyB/T training was dependent on characteristics of the training or group. Analyzing the idiographic items, 11 themes were found, covering various facets of mindfulness and mindful parenting, parental self-efficacy, maternal internalizing problems, self-care, and the parent-child relationship. All mothers showed improvement on multiple themes important to them personally. While one mother showed somewhat limited improvement on her idiographic items, for most mothers improvements with moderate to large effect sizes were found on most or even all themes covered by their idiographic items. Further, for some cases there appeared to be a discrepancy in results of the idiographic items and the corresponding nomothetic measures, with nomothetic measures appearing less sensitive. However, looking at the participants as a group, the majority of the group showed improvement on the predetermined nomothetic outcomes. Regarding practice quantity, large effect size correlations were found between minutes meditated per day and several mindfulness facets, mindful parenting and parental stress. No trends were found in effectiveness depending on training or group characteristics.

Regarding the first aim of the study, it is interesting to note that there was a strong agreement notable between mothers on some of the idiographic themes. With respectively 10, 9, and 11 items created regarding the themes mindfulness: emotional nonreactivity, mindful parenting: attention, and self-care, by seven to eight out of the ten participants, these were the most common themes in our sample. Boekhorst et al. (2021) found similar themes to be common among mothers of toddlers participating in an online mindful parenting training. These results indicate that interventions aimed at mothers of (very) young children could do well by including these topics.

For the second aim of the study, the participants scored the idiographic items during a baseline, an intervention, a post-test, and a follow-up phase. The baseline phase could subsequently be used as control data to which the intervention phase, the post-test phase and follow-up phase were compared. Thereby we were able to control for baseline scores and confounding variables. Using visual analysis, we found increasing trendlines for 86% of the participants during the intervention phase, compared to 65% of the participants during the baseline phase. Looking at differences in mean scores between phases per item, an improvement was found in most items, when comparing the intervention phase, post-test phase, and follow-up phase to the baseline phase (84%, 86%, and 94% respectively). Looking at differences in mean scores between phases per theme, improvement was found comparing later phases to baseline for all themes. Using NAP-scores, all mothers showed improvement and most mothers improved on most (or all) of their idiographic items. It should be noted that previous studies have indicated that many interventions do not have a significant effect on a majority of their participants (e.g., Cuijpers et al., 2021). In this light the results on the idiographic items are indeed very promising, MwyB/T appears to fit well with the complaints and treatment goals mothers of young children have.

Further, to triangulate our findings, we compared results on the idiographic items with results on corresponding validated nomothetic measures. Effects on the idiographic themes were often but not always confirmed by reliable changes on these corresponding nomothetic measures. Directly after the training, all mothers but one reported change on at least one theme that they identified as important for them. Changes were mostly seen on the area of mindfulness, and mindful parenting (especially listening with full attention). As expected given the theoretical advantages of idiographic measures over nomothetic measures, nomothetic measures seemed to be less sensitive for change in our study than the idiographic items.

Interesting though, is the finding from our visual analysis. Although more participants experienced improvement during the intervention period, indicating an intervention effect, participants already appeared to experience an improvement on their idiographic items during the baseline phase. In the one or two weeks before baseline, the participants have had three different appointments: (1) a pre-intake with the mindfulness trainer of about 30 minutes, (2) an intake of about an hour with the mindfulness trainer and a psychologist (second author), and (3) an appointment of an hour with a psychologist (second author) or a master student working as a clinical intern (first author) during which the items were composed. As studies show that a large part of the effect of therapy is realized by non-specific factors (Cuijpers et al., 2012), it is possible that change in the participants was instigated simply by the quantity of pre-baseline contact. Also, the communication techniques of these professionals such as empathic listening and helping the participants to explore and express their emotions and experiences may have played a role in the baseline improvement.

A different possible explanation is that the creating and daily rating of the idiographic items itself had positive effects on the participants. Most of the participants consciously created goal-focused and not problem-focused daily items. This may have contributed to the baseline improvement. Goal setting is an effective way of achieving change as it provides direction and increases motivation (Locke & Latham, 2019). Further, many of the participants told the researchers that they found the daily items to be helpful for noticing what went well that day. Potentially, creating and rating idiographic items did not only affect baseline improvement, but also added to the effect of the training itself. This would explain why a higher percentage of participants improved on the nomothetic measures than in a previous study (Potharst, Leyland, et al., 2021). However, although many parenting interventions encourage parents to set goals, empirical evidence for the effect of goal setting in parenting interventions is inconclusive. On the one hand, Becker et al. (2018) found goal-setting to be an important predictor of engagement of families in mental health interventions. Better engagement, in its turn, is associated with better treatment outcomes (Becker et al., 2018). On the other hand, in a field experiment Van Aar et al. (2021) actually found no effect of approach oriented goals and only a limited effect of avoidance oriented goals on the effectiveness of a brief parenting intervention. Yet, the goals created in their study may have been less personally meaningful as they followed a preset format and were created after watching very short video’s about the intervention and goal setting itself. This may not have led to an increased engagement. While in our study the goals set were the result of extensive contact between the participants and the trainers and psychologists, during which participants formed a clear view of their complaints and goals. Future studies could compare nomothetic outcomes between a group that did create and rate idiographic goals in a similarly thorough way as the current study, and a group that did not rate nor create idiographic goals. This way, a possible additive effect of the creation of idiographic items and of daily measurements for the MwyB/T training could be studied.

The third aim of the study was to assess the effectiveness of the MwyB/T training on improving scores on predetermined nomothetic outcome measures regarding mindfulness, mindful parenting, parental self-efficacy, maternal internalizing problems, and parental stress. Looking at these predetermined variables assessed using nomothetic measures, we found a reliable improvement for 50–70% of the participants on the mindfulness facets acting with awareness, nonjudging of inner experience, and nonreactivity to inner experience. Regarding mindful parenting we found a reliable improvement for 50–80% of the participants on the total scale, and on the subscales listening with full attention and nonjudgmental acceptance of parental functioning. Finally, we found 50–60% of participants improved reliably on the variables parental self-efficacy, maternal internalizing problems, and parental stress. On most outcomes a similar or larger percentage of participants improved than in a study on only mothers of toddlers (Potharst, Zeegers, & Bögels, 2021). Further, while Potharst et al. (2017) found a delayed effect on parental stress in mothers of babies at eight week follow-up, participants in the current study already showed improvement at posttest. However, in general results did show an increase in effect size throughout the phases on both idiographic and nomothetic measures. Therefore, results were largely congruent to both previous studies (Potharst et al., 2017; 2021).

Fourth, the study aimed to examine the examine to what extent improvement was dependent on the quantity of practice, which would indicate a dose-response effect. While Potharst and colleagues (2019) found no dose-response effect between minutes meditated per day and parental stress, we did find large effect size correlations between minutes meditated per day and several mindfulness facets, mindful parenting and parental stress. Although most of these correlations were only borderline significant, this was probably due to limited power because of the small sample size. Limited power may also explain the sparsity of significant or borderline significant correlations between the time spent on informal mindfulness practice and mindful parenting practice on the one hand and outcomes on the other hand. Significant correlations, all with large effect sizes, were found between the percentage of days that the mothers meditated and several mindfulness and mindful parenting facets. The results imply that both the length of meditations, and the regularity with which the mothers meditated may have had a positive effect on the outcomes, and not only on mindfulness outcomes, but also on parenting outcomes.

Case-based research has some advantages over larger trials, one of which is that it is not only possible to study whether an intervention works, but also under what circumstances and for whom. Previous studies investigating the effectiveness of MwyB/T have not assessed this. The fifth and final aim of the study therefore was to examine whether effectiveness of MwyB/T was dependent on training or group characteristics, such as baby versus toddler groups and clinical versus preventative groups. Further, due to the Covid-19 outbreak during the data collection phase of this study, we had to adapt to physical restrictions and offer the MwyB/T training (party) online or individually or with the mothers partner. While preferably the training takes place face-to-face in a group of four to six mothers, these circumstances offered us the opportunity to examine whether the effectiveness was dependent on these training characteristics. We found no clear differences in effectiveness for any of the training or group characteristics. This suggests that MwyB/T is an effective intervention in all its forms (for mothers of both babies and toddlers, online and offline, in groups and individually, with and without a partner) and in different settings (both preventative and in a clinical setting).

There are some limitations to this study to take into consideration. First of all, with daily ratings extending over a period of months participants tend to skip ratings some days. This too happened for some participants in this study, resulting in missing data. The question remains whether data were indeed missing at random or if some mothers skipped the questionnaire on more difficult days, or in contrast on days with a lot of fun activities. Also, the length of the baseline phase was not randomized. We aimed at a 14-day baseline for all participants, but due to the intervention being a group training, some participants had a shortened baseline in order to be able to participate. Further, ideally one would see stable scores on items’ baseline phase. However, a large percentage of trendline slopes for the idiographic items were positive, thus indicating improvement. This complicates interpretation of results from later phases. It is possible creating the idiographic items had a positive effect even before the intervention started. In our current design we were unable to distinguish between possible effects from creating idiographic items, rating these items daily, and the training itself. Finally, as is true for most studies on parental psychological problems and interventions (Aktar et al., 2019), an important limitation to the current study is that the sample consisted solely of mothers and no fathers were included. While paternal psychological problems are an important area of research, there were several reasons not to include fathers. Although the intervention is written for parents of all genders, few men participate making including them not feasible There are some explanations for the absence of fathers in the training. On the one hand, men are less likely than women to seek professional help for mental health issues (Letourneau, Dennis, et al., 2012). Further, while women more often have a tend and befriend stress response due to which they have a need for group support (Nickels et al., 2017; Taylor et al., 2000; Turton & Campbell, 2005), men tend to prefer one-on-one instead of group support and prefer to seek help separately from their partner (Letourneau, Tryphonopoulos, et al., 2012). This preference is supported by the findings that men are less committed to group therapy than women and effectiveness of group therapy for men tends to be lower for men compared to women (Ogrodniczuk et al., 2004). On the other hand, including men could reduce positive effects the training has for women. In groups existing of women only, women tend to feel safer allowing them to talk more freely and more intimately (Greenfield et al., 2013; Moreno et al., 2005).

All in all, the results of the current study indicate that MwyB/T seems to be an effective intervention for women with young children experiencing mental health problems and/or parenting related stress or insecurity. All mothers showed at least some improvement on self-determined (parenting) problems or (parenting) goals, and the majority of mothers even showed improvement on most or all of these problems or goals. The improvements that were seen on personalized items were partly confirmed by standardized questionnaires that matched the themes of the personalized items. Effectiveness did not seem to depend on the training or group characteristics. However, improvement on outcomes did seem to depend on the amount of mindfulness practice during the training. Also, the creation of personalized items and daily rating of these items may have played positive role in the improvements mothers made. Therefore, it is important that more research is done on the role of goal setting and daily ratings of goals in parenting interventions.
